# Are our cities falling behind? A German perspective on the supply for year-round green exercise

**DOI:** 10.3389/fspor.2025.1598959

**Published:** 2025-06-19

**Authors:** Konrad Reuß, Christopher Huth

**Affiliations:** Institute of Sports Science, Chair of Sports- and Health Management, University of the Bundeswehr Munich, Neubiberg, Germany

**Keywords:** green exercise, urban planning, sports facilities, public health, meteorological conditions

## Abstract

Green exercise, defined as physical activity practiced in natural environments, has been recognized for its numerous health benefits, such as improvement of physiological and psychological well-being or social benefits. However, there is a decrease in green exercise, especially during winter. Adverse weather conditions, such as precipitation and cold weather, lead to this decrease. However, another factor might be found in the availability of adequate sports spaces and facilities for green exercise during those conditions. So far, the availability of suitable sports spaces and facilities for urban year-round green exercise, particularly under varying meteorological conditions, remains underexplored. This study aims to assess the supply and suitability of urban sports spaces and facilities for year-round green exercise in Germany on an individual and organizational level. A comprehensive online survey was conducted to assess the supply from an individual-level perspective as well as from an organizational-level viewpoint. Results indicate that the availability of sports spaces and facilities for urban year-round green exercise is rated as adequate on an individual level. However, there are significant variations depending on age, sports type, and individual connection to nature. On the organizational level, however, the suitability of existing sports spaces and facilities is rated low. The study found a strong correlation between perceived accessibility and the likelihood of engaging in green exercise, suggesting that better-designed and promoted facilities could enhance participation rates. Moreover, the lack of adequate courses and information about existing year-round green exercises poses additional barriers that must be addressed by urban planning and sports governance.

## 1 Introduction

Urbanization as a global phenomenon is understood as population growth and increased densification in built-up areas (1). Today, more than half of the world's population resides in cities, and an increase in urban population concentration is anticipated in the future. Side effects of urbanization include environmental hazards such as increased traffic and noise, air pollution, the urban heat island effect, and, in particular, the loss of green spaces. The rapid growth that cities are experiencing also creates health threats, ultimately leading to illness and disease states. These threats include social inequality, lifestyle-related risk factors, and a lack of physical activity (PA) (2). Physical activity can be understood as people's movement, acting, and performing within culturally specific spaces and contexts (3). Urban green spaces (UGS) provide these spaces and contexts, and therefore can mitigate those healthcare threats by offering a variety of positive health and social benefits for all age groups. This includes providing incentives for engaging in PA in the first place (4). Green exercise (GE) has been defined as PA practiced in nature (5, 6). This activity is considered to provide even greater health benefits compared to PA in other environments (5), and according to WHO (7), few other public health measures can provide the same benefits as GE. In recent years, the importance of GE has been increasingly emphasized, especially during the coronavirus pandemic (8, 9). The impact of COVID-19, which has led to various restrictions, including sports, has shown that many people feel a strong need to be outdoors and active (10). Besides the burden on the healthcare system caused by the pandemic, the German healthcare system already has the highest total expenditure in the healthcare sector within the European Union (11). GE can be seen as an accessible and cost-effective form of PA, which should therefore be considered an effective public health measure (4). However, even though GE is generally affordable and accessible, it is significantly affected by various factors (12). These factors include seasonal changes, daylight availability, and weather conditions such as temperature, wind, and precipitation (12–14). Adverse weather conditions, such as rain, cold, and wind, pose significant barriers and often lead to a considerable decline in physical activity (12, 14, 15). GE performed throughout the year might lead to profound health benefits. GE performed throughout the year, during all weather conditions, with moderate to vigorous intensity, will be referred to as year-round green exercise (YRGE). In addition to meteorological conditions, another barrier for GE can be seen in finding suitable spaces (16). Sports facilities are explicitly created for sports, including, for example, calisthenics parks. Sports spaces, sometimes referred to as sports opportunities, are considered areas that can be utilized for sports but were created for other purposes. These could include field and forest paths for running or lawns in UGS for bodyweight exercises or yoga, and are often used for informal sports. For this study, sports facilities are understood as areas where the primary use is intended to be through sports, whereas sports spaces have only a secondary use for sports. UGS, such as public parks and green areas, serve as vegetation sites, providing sports facilities and spaces with opportunities for GE (17, 18). Therefore, UGS play a crucial role in addressing public health challenges (19). Urban YRGE can increase physical activity levels and promote a more active lifestyle (20). In general, GE can lead to health benefits: psychological benefits, such as increased self-esteem, improved mood, and overall well-being; physiological benefits, such as reduction in blood pressure and reduced cardiovascular illnesses; cognitive benefits, such as restoring attention and reduced fatigue; social benefits, such as higher social cohesion and facilitated social connection; and spiritual benefits such as increased inspiration and spiritual well-being (21). These benefits can be found regardless of age, sex, and socio-economic status (22, 23).

Various factors influence YRGE. These include seasonal aspects such as the amount of daylight and weather conditions like temperature, wind, or precipitation (14), as well as the availability of suitable sports facilities or spaces that support outdoor activities under different seasonal conditions (16). According to the theory of planned behavior, one's attitude towards the behavior, the subjective norm, and the perceived behavioral control influence the actual behavior (24). In the context of YRGE, suitable sports spaces and facilities, and one's attitude towards GE and nature-relatedness can strongly influence the exact amount of urban YRGE, especially during adverse weather conditions.

This study assesses the prerequisites for urban YRGE during different meteorological conditions. Therefore, the research questions for this study are as follows: First, what does the current supply of suitable sports spaces and facilities for urban YRGE look like from the perspective of an individual level as well as from the perspective of cities and sports organizations? Second, are sufficient sports offers provided on an organizational level, and how do the organizations rate the benefits of urban YRGE? To the author's knowledge, the supply of sports facilities and spaces for urban YRGE in different meteorological conditions has not been investigated. This study contributes to closing this gap by examining the current supply of sports facilities and spaces for urban YRGE. The article is structured as follows: The methodology will be described after this introduction. Subsequently, the results will be presented and discussed, followed by the conclusion and future research questions.

## 2 Materials and methods

The data was collected via an online survey using the SoSci Survey platform. It was recorded on an individual and an organizational level to understand supply and demand better. For a holistic understanding, two questionnaires were created. One questionnaire was aimed at people living in urban areas and practicing GE, and the other was aimed at organizational representatives. The questionnaires were designed to record the ratings of existing sports spaces and facilities and the courses offered for practicing YRGE. In addition to the infrastructural conditions and other aspects that can influence GE, the perception of sports spaces and facilities as suitable places for GE during different meteorological conditions was also recorded. Since GE is strongly reduced during meteorological conditions such as rain, snowfall, or cold, some of the questions were explicitly aimed at meteorological conditions that are generally perceived as bad weather (15). The participants received a brief explanation for the different weather conditions. For example, cold was referred to as below zero degrees, heat was above 30 degrees, and strong wind was defined as more than 40 km/h.

For the distribution of the questionnaire, state sports associations and sports federations, as well as major German cities with a population of more than 100,000 inhabitants, were contacted proactively. They were then asked to forward the questionnaire to clubs or other providers for urban GE. The participant's recruitment from the individual's perspective was based on the snowball principle; no eligibility criteria were applied, and no incentives for participation were provided. Both questionnaires were available online from January 15, 2024, to March 15, 2024. Both questionnaires were available in German and had been tested beforehand for clarity of wording and logical structure, and revised accordingly. The internal consistency of the individual question blocks was checked using Cronbach's alpha. For both questionnaires, where possible and appropriate, a 5-point Likert scale with (1) disagree to (5) agree was used to answer the questions. These scales have been widely used since they best reflect the participants' perspectives (25, 26). The organizations were also asked to state their zip code. A total of 177 questionnaires were completed at the organizational level, of which 93 were ultimately used for the evaluation. Questionnaires with missing data were excluded from the analysis. Table 1 provides an overview of the questionnaire structure for the organizational participants, consisting of one block of questions regarding the demand and supply of urban YRGE. The demand was recorded using the question, “How do you assess the demand for urban year-round green exercise?”. Four items (*α* = .77) assessed the existing supply, with questions such as “In your opinion, is there a sufficient supply of sports spaces and facilities for year-round green exercise?” being asked. Furthermore, perceived advantages or downsides from YRGE were assessed by asking questions such as “What advantages do you see in practicing urban green exercise, especially in bad weather?”

**Table 1:** Structure of the questionnaire for the organizational level representatives and a brief description of items.

Item	Description	Level of scale
Demand for YRGE	Organizational-level representatives were asked to estimate the level of demand for YRGE	Ordinal
Suitable sports infrastructure	Organizational-level representatives were asked to rate the suitability of existing sports spaces and facilities for YRGE	Ordinal
Benefits and downsides	Organizational-level representatives were asked to state whether there are any benefits or downsides to YRGE	Ordinal
Organizational information	Organizational-level representatives were asked to state their affiliation	Nominal

A total of 772 questionnaires were completed for the perspective on the individual level, of which 408 could ultimately be used for the evaluation. Questionnaires with missing data were excluded from the analysis. The questionnaire for the individual-level participants consisted of multiple sections. Table 2 provides an overview of the structure. The first section included questions on GE, such as “What sports do you do most often outdoors in urban green spaces?” (27). The following section dealt with GE during different meteorological conditions. Those conditions were (a) precipitation, (b) slippery or muddy ground, (c) extreme cold, (d) frozen ground, (e) strong wind, (f) extreme heat, and (g) strong sun radiation. GE during different meteorological conditions was recorded with eight items (*α* = 0.87), which included questions such as “How likely are you to be active outdoors even in precipitation (rain or snowfall)?”. Furthermore, this section dealt with the perceived availability of suitable sports spaces and facilities for YRGE during different meteorological conditions by providing statements such as “There are enough suitable sports facilities” (28). This section also included questions about the availability of courses, such as guided fitness programs or municipal initiatives, for YRGE. The NR-6 scale (*α* = 0.82) was used in the final section to measure the general individual connection to nature. The NR-6 scale comprises six statements, such as “I perceive the animal world wherever I am.” The NR-6 score was calculated by averaging all six items. The higher the score, the more pronounced the connection to nature (29).

**Table 2:** Structure of the questionnaire for the individual level participants and a brief description of items.

Item	Description	Level of scale
Demographics	Participants were asked to state their age, gender, level of education, income, profession, and residency	Nominal and interval
GE during different meteorological conditions	Participants were asked to state the possibility of being active during different meteorological conditions	Ordinal
Sports spaces and facilities	Participants were asked to rate the suitability of the existing sports spaces and facilities of YRGE	Ordinal
Nature relatedness	Participants were asked to answer the NR-6 scale to reveal their connection to nature	Ordinal

The data was initially analyzed descriptively. A series of Spearman rank correlations and Pearson's correlation were calculated for the statistical calculation of the correlations. ANOVA calculated differences between groups with Bonferroni *post hoc*, and effect sizes were calculated. The data from the questionnaires were analyzed using IBM SPSS Statistics Version 29.0.2.

## 3 Results

The result section begins with a description of the participant's characteristics. It then follows the perspective of the individual level on the supply of adequate sports spaces, facilities, and courses offered by urban YRGE. The perspective from cities and sports organizations follows, including expected benefits from urban YRGE.

### 3.1 Participants' characteristics

Of the individual-level participants, 53.9% (*n* = 220) are female, and 46.1% are male (*n* = 188). The average age of the participants is between 41 and 50 years, and the average net monthly income is between € 2,001 and € 3,000. Regarding educational level, 22.7% (*n* = 93) stated that their highest level of education was secondary school, intermediate school, or A-level. 58.6% (*n* = 239) indicated that they had a university degree (bachelor's, master's, doctorate, etc.), 15.7% (*n* = 64) indicated that they had completed vocational training, and 3.0% (*n* = 12) indicated that they were still at school or had no school-leaving qualification. 59.3% (*n* = 242) stated that their work is predominantly sedentary, 19.1% (*n* = 78) are retired, 14.7% (*n* = 60) do physically demanding work (e.g., haulage, construction site, etc.) or manual labor (e.g., electrical, plumbing, carpentry, etc.), and 3.9% (*n* = 16) have a job that is mainly outdoors (e.g., gardening, etc.). 96.3% (*n* = 393) of the participants live in Germany.

**Figure 1:**
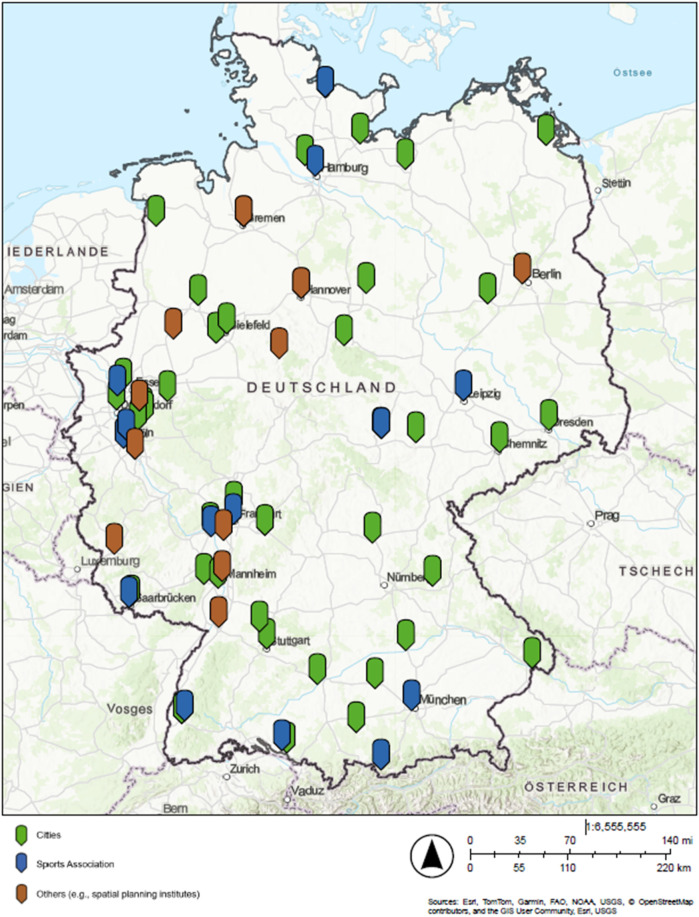
Geographical distribution of the organizational level participants.

Of the 93 participants in the survey for the organizational level, 50.5% (*n* = 47) are cities. Of these, 22.6% (*n* = 21) are cities with more than 100,000 inhabitants, and 16.1% (*n* = 15) have a population between 100,000 and 20,000. 32.3% (*n* = 30) of the participants belonged to sports associations. These included 11 federal state sports associations and four governing sports associations. The remaining 17.2% (*n* = 16) included other organizations, such as institutes for sports space planning or institutes for sports facility development. Based on zip code, the geographical distribution (Figure 1) of the organizations in Germany shows an imbalance between the southwest and the northeast. 38.7% (*n* = 36) of the organizations are from the south, 30.1% (*n* = 28) are from the west, whereas 16.1% (*n* = 15) are from the north, and 8.6% (*n* = 8) are from the east of Germany.

Climatic conditions in Germany vary significantly between the northeast and the southwest, shaped by geographical location and regional topography. The southwest, influenced by Atlantic air masses and the sheltering effect of the Central Uplands, generally experiences milder and warmer temperatures compared to the northeast. Summers in the southwest tend to be warmer, while winters are less severe. In contrast, the northeast exhibits a more continental climate, with greater temperature extremes and colder winters. Precipitation patterns also differ: the southwest receives more annual rainfall, especially in orographically influenced regions, whereas the northeast is among the driest parts of the country, with frequent summer droughts. Regarding solar radiation, northeastern Germany records higher annual sunshine durations. Lastly, wind conditions are more pronounced in the northeast due to the flat landscape and proximity to the Baltic Sea, which enhances the impact of easterly and northeasterly winds, especially during the winter months. Conversely, the southwest is more sheltered and typically less windy (30).

### 3.2 Supply of adequate sports spaces

**Figure 2:**
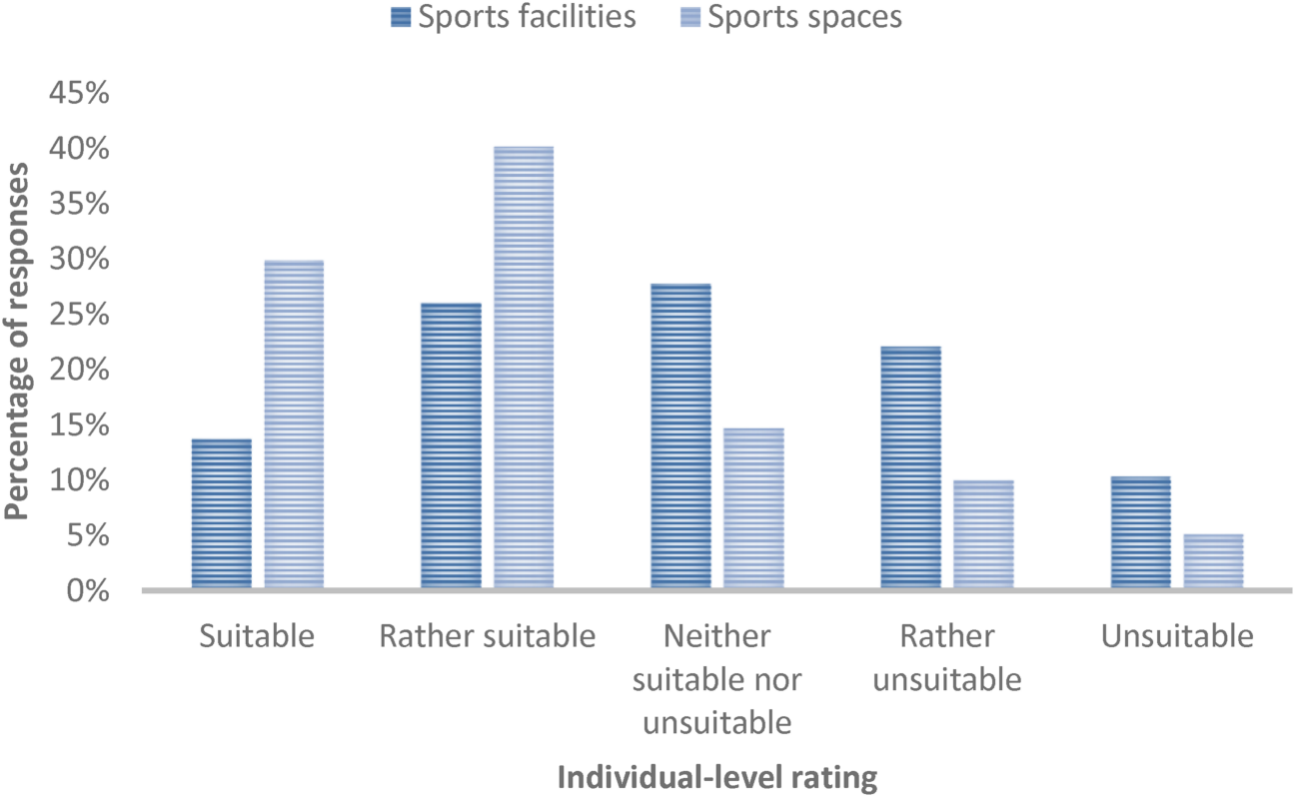
Rating of the suitability of sports spaces and facilities on an individual level.

The suitability of sports spaces for physical exercise has a mean score of *µ* = 3.8 (SD = 1.12). Overall, 29.9% (*n* = 122) and 40.2% (*n* = 164) consider the existing sports spaces to be suitable or somewhat suitable for being active during adverse weather conditions, 14.7% (*n* = 60) consider them neither appropriate nor unsuitable, 10.0% (*n* = 41) consider them rather unsuitable, and 5.1% (*n* = 21) consider them unsuitable. Figure 2 provides an overview of these findings.

A significant positive correlation (*p < .05, **p < .01) between the presence of suitable sports spaces and the probability of being physically active in meteorological conditions (a) precipitation *r* = .216** (*η*² = .047), (b) slippery or muddy ground *r* = .186** (*η*² = .035), (c) extreme cold *r* = .217** **(***η*² = .047), (d) frozen ground *r* = .107* (*η*² = .011), (g) strong sun radiation *r* = .120** (*η*² = .014) was found (Table 3). In addition, a significant positive correlation exists between an activity of at least 5 min and the availability of suitable sports spaces (*r* = .195**, *η*² = .038). Effect sizes for these findings range from small to medium. For the assessment of the suitability of sports spaces, there is a significant positive correlation with the frequency of visiting UGS (*r* = .275; *p* < .01). A significant difference [F(6,401) = 5,650, *p* < .001] in the assessment of the suitability of the spaces for GE in adverse weather conditions between the sports clusters was found. The endurance sports (*µ* = 4.04; SD = 1.02) and gymnastic sports (*µ* = 3.96; SD = 1.26) were perceived as more suitable, while strength sports (*µ* = 3.19; SD = 1.26) were perceived as less suitable. The effect size (*η*² = .078) suggests a medium-sized influence of sport type on the perception of green space availability. A significant difference was also found between the age groups [F(4,401) = 5,713, *p* < .001]. Younger people aged >20 years (*µ* = 3.53; SD = 1.26) and 21–30 years (*µ* = 3.50; SD = 1.20) tend to perceive UGS as more unsuitable, and elderly people aged 61–70 years (*µ* = 4.18; SD = .90) and 81 years and older (*µ* = 4.25; SD = .93) tend to see them as more suitable. However, the effect size is small (*η*² = .047). For the remaining socio-demographic factors—gender, income, education, and nature-relatedness—no significant differences were found in assessing the suitability of sports spaces.

**Table 3:** Spearman-Rho correlation between the perceived suitability of sports spaces and facilities and the probability of being active during different meteorological conditions.

Item	Precipitation	Muddy ground	Extreme cold	Frozen ground	Strong wind	Extreme heat	Strong sun radiation
Spaces	.216**	.186**	.217**	.107*	.068	.064	.120*
Facilities	.193**	.141**	.153**	.116*	.100*	.091	.163**

* *p* < .05; **p < .01.

### 3.3 Supply of adequate sports facilities

The suitability of the sports facilities has a mean score of *µ* = 3.11 (SD = 1.19). In total, 13.7% (*n* = 56) saw these as suitable, 26.0% (*n* = 106) saw them as rather suitable, 27.8% (*n* = 114) as neither suitable nor unsuitable, 22.1% (*n* = 90) saw them as rather unsuitable, and 10.3% (*n* = 42) saw them as unsuitable. Figure 2 provides an overview of these findings. The presence of suitable sports facilities correlates significantly positively (*p < .05, **p < .01) with the probability of being physically active in meteorological conditions (a) *r* = .193** (*η*² = .037), (b) *r* = .141** (*η*² = .020, (c) *r* = .153** (*η*² = .023), (d) *r* = .116* (*η*² = .013), (e) *r* = .100*, *η*² = .010) (Table 3). Additionally, a significant positive correlation exists between being active for at least 5 min and the availability of suitable sports facilities (*r* = .163**, *η*² = .027). There is also a significant positive correlation between the assessment of the suitability of the sports facilities and the frequency of visits to UGS (*r* = .140; *p* < .01, *η*² = .020). Effect sizes for these findings tend to be small.

A significant difference in the perceived suitability of the sports facilities and age [F(7,400) = 4,283, *p* < .001] with a small to medium effect (*η*² = .070) was found. Younger people aged 21–30 years tend to perceive the facilities as less suitable (*µ* = 2.69; SD = 1.20), whereas elderly people aged 61–70 years (*µ* = 3.51; SD = 1.00) and those 81 years and older (*µ* = 3.81; SD = 0.83) tend to perceive them as more suitable. No significant differences were found in the suitability of the facilities and sports clusters, as well as in socio-demographics, gender, income, and education. A significant positive correlation was found between connection to nature and the assessment of suitable sports facilities (*r* = .174; *p* < .01), with a small effect size (*η*² = .030).

### 3.4 Offers from organizations and options for self-organized activities

On an individual level, 44.2% (*n* = 180) rate the existing offers from sports associations as (rather) sufficient, 32.4% (*n* = 132) see them as neither sufficient nor insufficient, and 23.6% (*n* = 96) see them as (in)sufficient. The existing commercial offers are rated as (rather) insufficient by 35.8% (*n* = 148), 41.2% (*n* = 168) rate these as neither sufficient nor insufficient, and 22.5% (*n* = 92) rate these as (rather) sufficient. For self-organized activities, 73.0% (*n* = 297) rate the existing options as (rather) suitable, 15.4% (*n* = 63) see them as neither suitable nor unsuitable, and 11.7% (*n* = 48) see them as (un) suitable. No differences in the perceived suitability of associations and commercial offers could be found between the sports clusters and socio-demographics. A significant difference was found in the options for self-organized activities between the sports clusters [F(6,401) = 3.58, *p* = .002], with the endurance cluster (*µ* = 4.33, SD = 1.78) rating the existing options as better than the strength cluster (*µ* = 3.47, SD = 3.47). A significant positive correlation was found between connection to nature and opportunities for self-organized exercise (*r* = .162; *p* < .01).

At the organizational level, 51.6% (*n* = 48) advertise the existing offers for urban YRGE on their websites. Other options, such as mailing lists (11.8%, *n* = 11), flyers or magazines (10.8%, *n* = 10), or information events (8.6%, *n* = 8), are also used.

### 3.5 Perspective from a sports organizational level on urban YRGE

**Figure 3:**
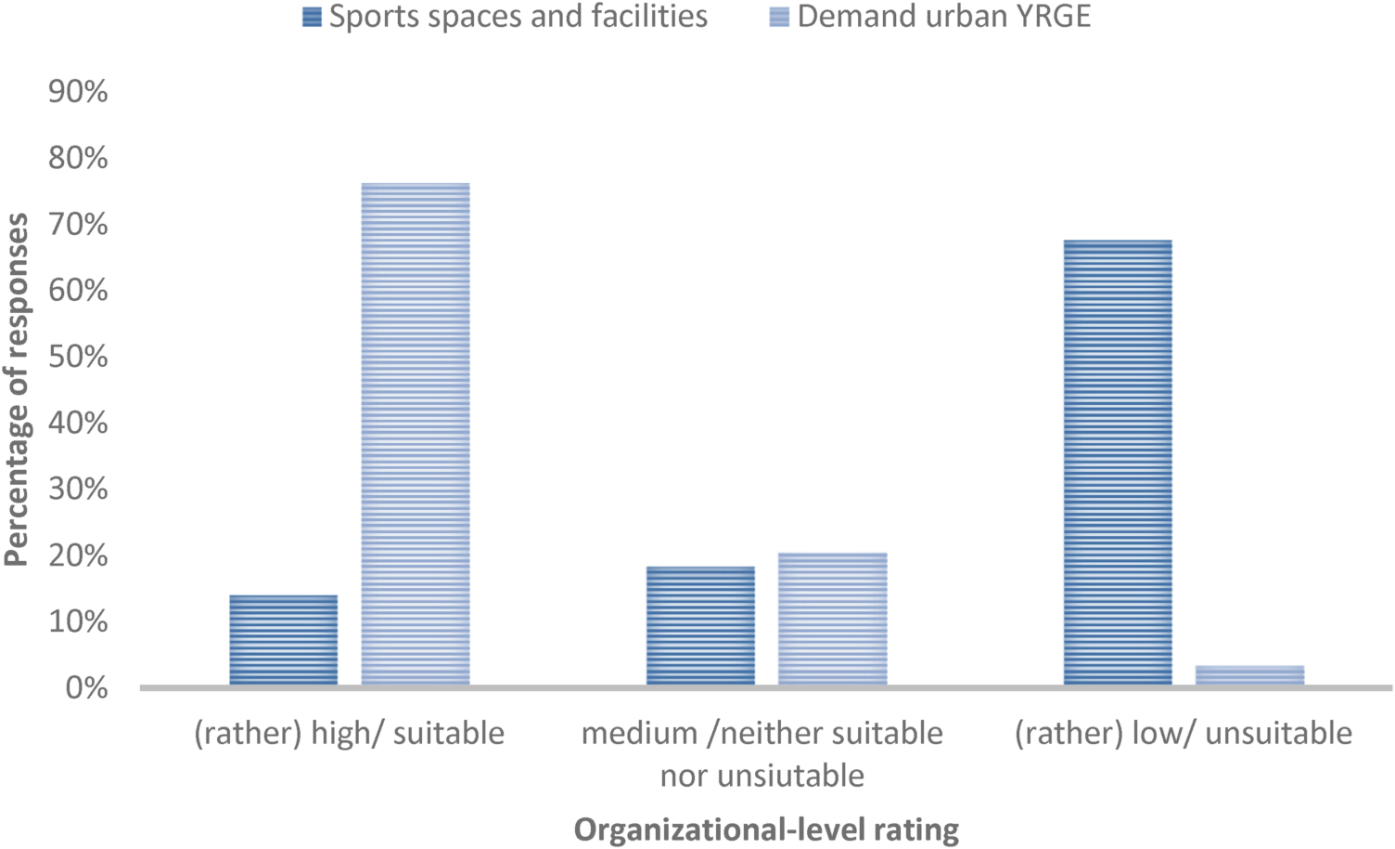
Organizational-level rating for demand for urban YRGE and the suitability of sports spaces and facilities.

The demand for urban YRGE is rated as (very) high by 76.3% (*n* = 71) from the organizational level perspective, 20.4% (*n* = 19) rated it as medium, and 3.3% (*n* = 3) consider it to be (very) low. The existing sports spaces and facilities are considered to be (rather) unsuitable for urban YRGE by 67.7% (*n* = 63), 18.3% (*n* = 17) consider these to be neither suitable nor unsuitable, and 14.0% (*n* = 13) rate these as (rather) suitable. The existing offerings for urban YRGE courses are rated as (rather) insufficient by 62.4% (*n* = 58), 23.7% (*n* = 22) rate them as neither insufficient nor sufficient, and 11.9% (*n* = 11) consider them to be (rather) sufficient. Figure 3 provides an overview of these findings.

There is no difference in the assessment of the sports spaces, facilities, and course offerings between the different types of organizations or regarding their geographical location. However, there is a significant difference between the assessment of the suitability of sports spaces and facilities for GE in adverse weather conditions on an individual level and organizational level [F(1,499) = 18,969, *p* < .001]. The organizational level (*µ* = 2.95; SD = .96) tends to rate the suitability of sports spaces and facilities worse than the individual level (*µ* = 3.45; SD = 1.02).

Table 4 provides an overview of the main findings from the individual and organizational perspectives.

**Table 4:** Overview of the main findings from the individual and organizational perspectives.

Item	Individual level	Organizational level
Supply of suitable sports spaces	(rather) low for strength sports and younger people, somewhat sufficient for endurance sports and elderly people	(rather) low but (very) high demand for YRGE
Supply of suitable sports facilities	(rather) low but more suitable for elderly people	(rather) low but (very) high demand for YRGE
Course offers	insufficient offers, but self-organized YRGE is possible	insufficient advertisement

### 3.6 Organizational-level view on the benefits resulting from urban YRGE

**Figure 4:**
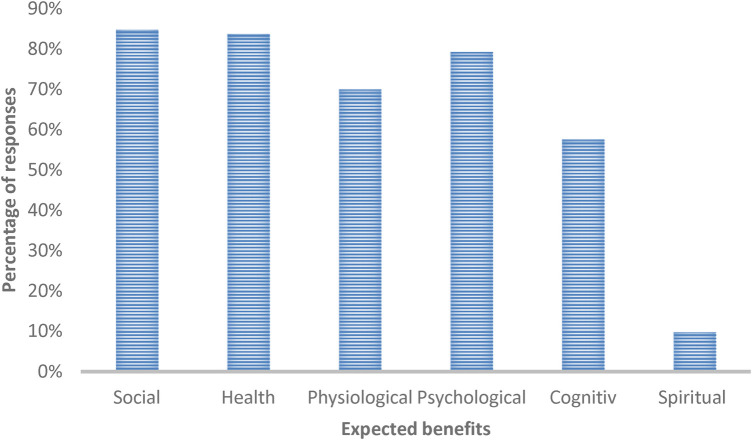
Organizational-level perspective on the expected benefits resulting from urban YRGE.

From an organizational level's perspective, the benefits resulting from YRGE are social benefits (84.8%, *n* = 78) and overall health benefits (83.7%, *n* = 77), such as improved immune defense or fall prevention. Urban YRGE, especially in adverse weather conditions, is not perceived as a risk of increasing injuries by 49.5% (*n* = 46) of sports policy representatives. In contrast, the remaining 50.5% (*n* = 47) perceive an increased risk of injury. 66.7% (*n* = 62) see no increase in the risk of illness, and 93.5% (*n* = 87) do not see any other disadvantages resulting from urban YRGE. Furthermore, 70% (*n* = 64) see physiological benefits such as improved endurance or strength, and 79.3% (*n* = 73) see psychological benefits such as enhanced well-being. Cognitive benefits, such as improved concentration, are reported by 57.6% (*n* = 53), and spiritual benefits, including a more profound sense of connectedness, are reported by 9.8% (*n* = 9) as results from the urban YRGE. An overview of these findings is presented in Figure 4.

Different groups of people use UGS for various activities. However, 80.6% (*n* = 75) of the organizations do not see any increased potential for conflicts with urban YRGE users in the same spaces.

## 4 Discussion

This study's key finding is that the supply of sports spaces for urban YRGE seems sufficient on an individual level, whereas the supply of sports facilities is rated as less sufficient. On the other hand, the representatives at the organizational level seem to rate the existing sports spaces and facilities as insufficient while stating that the demand for YRGE is perceived as high. This result must still be interpreted cautiously since the distribution of cities and organizations throughout Germany is skewed. Furthermore, differences in climate throughout Germany might influence the perception of YRGE. Different regions throughout Germany experience adverse weather conditions. The south-west might experience milder winters but tends to have more precipitation. The northeast tends to experience colder temperatures, stronger winds, especially during the winter, and more annual solar radiation. Given these differences, sports spaces and facilities should be designed to accommodate regional climatic conditions, enabling people to stay active under various meteorological conditions. The probability of being active during adverse weather conditions suggests that, on an individual level, those who are already active perceive sports spaces as more suitable, whereas others may not. Furthermore, the connection to nature can influence the perception of the suitability of sports facilities; however, since the effect size is small to medium, this result needs to be interpreted cautiously, and future studies should investigate the role of nature connectedness in the context of YRGE. The difference in perception between different sports clusters shows potential differences in perceived behavioural control, which influences the likelihood of behavioural achievement (24). The supply of adequate sports facilities for urban YRGE is rated as insufficient compared to that of sports spaces. However, the differences in the rating between groups indicate that there might be a big difference depending on the sports practiced. For example, the existing sports spaces seem to favour endurance sports, and older people tend to rate the supply of sports spaces and facilities as more sufficient. The more positive rating of the elderly can be regarded as a good thing, since the elderly greatly benefit from GE (31). However, since the differences between the age groups only had a small to medium effect, this might indicate that facilities and spaces for YRGE are perceived similarly by different groups. Therefore, this supports the statement that GE is an accessible form of PA for all (5), which also appears to be true for YRGE. Nonetheless, increasing the amount of GE in vulnerable groups can be beneficial in reducing non-communicable diseases (32), which can lead to lowering the high costs of the German healthcare system. However, since the results of this study are inconsistent regarding the supply of adequate sports spaces and facilities, finding suitable sports spaces and facilities can still be a potential barrier for GE (16). Adequate sports facilities don't need to be new installations, rebuilding existing facilities may already be beneficial (33). Especially since the availability and accessibility of UGS in Germany already appear to be very good (34, 35), the suitability of sports spaces and facilities for urban YRGE needs to be improved. Therefore, future studies should investigate which characteristics of UGS are essential for people to engage in YRGE. However, since the distribution of organizations and cities participating in this survey shows an imbalance from northeast to southwest, this may skew the results. Therefore, caution is required when interpreting these results.

The results from this study also indicate that nature-relatedness seems to influence the perception of the suitability of sports spaces and facilities on an individual level. However, the findings regarding the influence of nature-relatedness are inconsistent since the rating of sports facilities correlated with nature-relatedness, whereas the rating for sports spaces did not. Previous studies have shown a tendency for a correlation between nature-relatedness and YRGE (35), which also appears to hold for assessing the suitability of the spaces and facilities. The findings from this study suggest that an individual's relationship with nature can play a role in influencing their perceived behavioral control. Therefore, future studies should assess the influence of nature-connectedness on using sports spaces and facilities for YRGE.

Another finding from this study is that from an organizational-level perspective, the majority recognize the potential health benefits (21) resulting from urban YRGE and don't see any significant disadvantages. This leads to the conclusion that urban YRGE is considered a good health measure, which aligns with the WHO's perspective on GE (7) and supports previous findings on health benefits resulting from GE (1, 21). Although this was not the primary focus of the study, the organizational perspective on general health benefits provides additional information about YRGE, emphasizing the need to promote YRGE. However, this aspect of the YRGE phenomenon warrants further investigation in future studies. Findings from this study further demonstrate the lack of suitable courses for urban YRGE. The existing courses offered for urban YRGE are rated as inadequate overall, which can be seen as a potential barrier. Providing adequate courses for GE during different meteorological conditions can encourage the general population to engage in more PA (15). However, there might also be a lack of knowledge about existing offers for urban YRGE, as only around half of the organizations provide information about existing offers on their websites, and only a very few organizations use other forms. The lack of information and knowledge has already been identified as a key reason why people do not visit green spaces (36, 37), and this could also be a barrier to engaging in urban YRGE. However, since most urban YRGE is self-organized, the lack of adequate courses needs to be addressed by the sport's governance in the future. Getting more people to engage in GE can address specific threats posed to society (2), which is confirmed by the sports organizational view on potential social, physiological, and psychological benefits from urban YRGE.

Due to certain limitations of this study, the results should be interpreted with caution. One limitation of this study is that the participants already tend to engage regularly in YRGE, and most are involved in endurance sports such as running or Nordic walking. The NR-6 scale score in this survey indicates that the participants' connection to nature seems to be reasonably strong. The NR-6 score for the participants on an individual level has an average of 3.6 (SD = 0.8). Therefore, this might limit the generalizability of the findings. Regarding the socio-economic characteristics of the participants, it is worth noting that their educational level is relatively high. Furthermore, the youngest (<20 years) and oldest (>60 years) participants tend to be underrepresented, which might also be seen as a limiting factor that might skew the results and therefore limit generalizability. Another limitation is that the sports practiced were predominantly endurance sports. It may also be questionable whether participants were aware of the distinction between a sports facility and a sports space, despite examples of each being provided in the questionnaire. Since sports facilities and spaces encompass a broad variety, ranging from non-equipped areas to properly designed facilities, there is a potential that participants might not be able to correctly distinguish between these types when considering the areas they use for their personal YRGE. Furthermore, using online surveys as a convenience sampling method might limit the generalizability of this study's findings. Therefore, future studies should aim to address the remaining gaps by considering these limitations.

## 5 Conclusions

Since there seems to be a high demand for urban YRGE (35) and a simultaneously inadequate supply, there is a need to improve the existing sports spaces and facilities. In addition, enhancing the offerings of courses from sports associations for urban YRGE and improving the advertising of existing courses, spaces, and facilities can increase the amount of YRGE. This can be seen as a valuable and easy-to-establish measure and is therefore highly recommended for implementation by sports organizations and cities. Since urban YRGE appears to lead to positive health benefits, a general improvement in sports facilities and spaces for urban YRGE may further enhance these effects. The availability of suitable sports spaces and facilities can support the urban YRGE. Therefore, the design of UGS and sports facilities is crucial for promoting urban YRGE. This increase in PA reduces public health costs (38). As a result, the government should mobilize to invest more in the required sports infrastructure. Establishing adequate sports spaces and facilities for urban YRGE for different sports can reinforce GEs' ability to function as a cost-effective and accessible health measure for all (4). However, the mere presence of sports facilities is not enough to encourage people to be physically active (4). Nature-relatedness has been identified as a moderating factor for YRGE (35). Therefore, sports managers and politicians should aim to find ways to increase nature-relatedness, which could be achieved by providing more suitable sports. These spaces lead to people spending more time in nature, which may increase their connection to nature (39), eventually leading to more urban YRGE.

## Data Availability

The raw data supporting the conclusions of this article will be made available by the authors, without undue reservation.
