# Correction: Are our cities falling behind? A German perspective on the supply for year-round green exercise

**DOI:** 10.3389/fspor.2025.1654443

**Published:** 2025-07-30

**Authors:** Konrad Reuß, Christopher Huth

**Affiliations:** Institute of Sports Science, Chair of Sports- and Health Management, University of the Bundeswehr Munich, Neubiberg, Germany

**Keywords:** green, exercise, urban, green space (GS), YRGE

The funder Universität der Bundeswehr München was erroneously omitted.

The original version of this article has been updated.

